# Author Correction: Hapten-mediated recruitment of polyclonal antibodies to tumors engenders antitumor immunity

**DOI:** 10.1038/s41467-021-25400-1

**Published:** 2021-11-22

**Authors:** Brett Schrand, Emily Clark, Agata Levay, Ailem Rabasa Capote, Olivier Martinez, Randall Brenneman, Iris Castro, Eli Gilboa

**Affiliations:** Department of Microbiology & Immunology, Dodson Interdisciplinary Immunotherapy Institute, Sylvester Comprehensive Cancer Center, Miller School of Medicine, University of Miami, Miami, FL 33136 USA

**Keywords:** Cancer, Cancer therapy, Cancer immunotherapy, Tumour immunology, Immunology

Correction to: *Nature Communications* 10.1038/s41467-018-05566-x, published online 22 August 2018.

This Article contained an error in the Introduction and Discussion sections. The original version of the Article omitted references to additional studies that have used the concept of ligand targeted recruitment of antibodies.

In the Introduction, we have added references to these studies and the second sentence of the third paragraph now reads ‘Spiegel and colleagues have shown that DNP conjugated to a PSMA-binding small molecule recruits DNP-specific antibodies to PSMA expressing LnCAP tumor cells, mediates their lysis in an ADCC dependent manner and inhibits LnCAP tumor growth in DNP vaccinated humanized mice (22, 23). Investigators at Altermune Inc. have conjugated an alpha-gal trisaccharide (agal), a hapten that binds to naturally occurring antibodies in humans, to a DNA aptamer that binds to the surface-anchored M protein of group A Streptococcus (GAS), and showed that binding of the GAS-agal conjugate to Streptococcus bacteria recruits anti-agal antibodies from human sera to the bacteria that are taken up and killed by human phagocytes (24).’

In the Discussion, the following sentences have been added to the end of the third paragraph: ‘The concept of recruiting antibodies to cell surface with ligand targeted antigens has been previously described (21-24). Here we describe a broadly applicable, if not universal, drug formulation to coat tumor cells in vivo with polyclonal antibodies whereby naturally occurring ubiquitously present antibodies are recruited to tumor cells in mice using a pan-cancer VEGF targeting ligand conjugated to an aGAL trisaccharide, and show that it leads to the regression of highly aggressive B16BL6 tumors (Figure 6).’ Reference 21 was already cited in the manuscript and the correction adds references to it. The following references have been added to reflect these changes:

22. Murelli, R. P., Zhang, A. X., Michel, J., Jorgensen, W. L., & Spiegel, D. A. Chemical control over immune recognition: a class of antibody-recruiting small molecules that target prostate cancer. *J. Am. Chem. Soc*. **131**, 17090–17092 (2009).

23. Dubrovska, A. et al. A chemically induced vaccine strategy for prostate cancer. *ACS Chem. Biol*. **6**, 1223–1231 (2011).

24. Kristian, S. A. et al. Retargeting pre-existing human antibodies to a bacterial pathogen with an alpha-Gal conjugated aptamer. *J. Mol. Med.* **93**, 619–631 (2015).

References numbers have also been updated in light of these additions. These errors have now been corrected in the HTML and PDF version of the Article.

