# Supplementary material for: Hapten-mediated recruitment of polyclonal antibodies to tumors engenders antitumor immunity
Source: Nat Commun. 2018 Aug 22;9:3348. doi: 10.1038/s41467-018-05566-x (PMC6105580; doi:10.1038/s41467-018-05566-x)
Supplement: Supplementary file 1 — Supplementary Information [file 41467_2018_5566_MOESM1_ESM.pdf]

SUPPLEMENTARY FIGURES:

Hapten-mediated recruitment of polyclonal antibodies to tumors engenders antitumor immunity

Schrand et al.

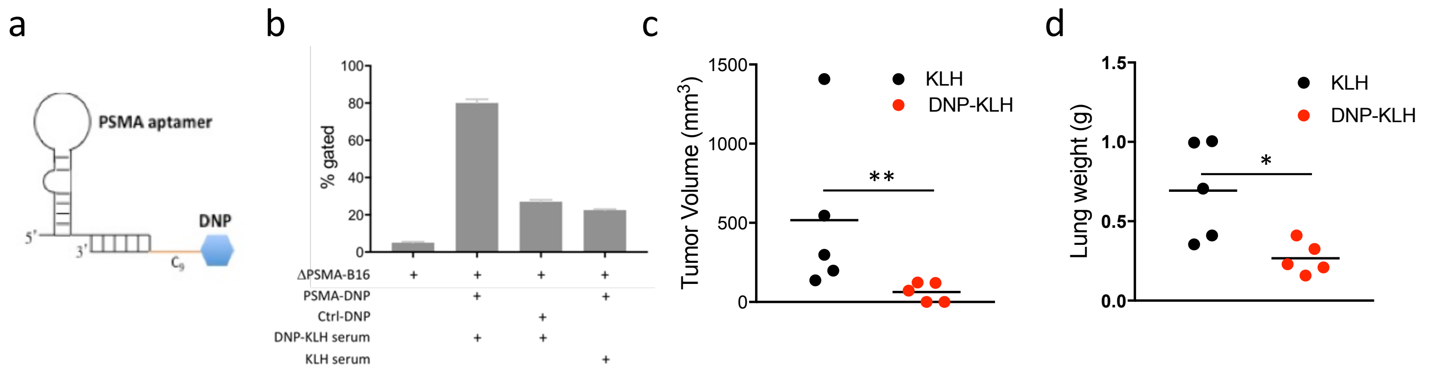

Supplementary Figure 1. Targeting DNP to prostate tumor cells inhibits tumor growth in DNP immune mice. **a.** A human PSMA aptamer conjugated to DNP by hybridization via short complementary sequences as described in Methods. **b**. B16.F10 tumor cells engineered to express a mutant PSMA that does not internalize upon PSMA aptamer engagement[23] (ΔPSMA-B16) was incubated with DNP-KLH or KLH immune sera in the presence of either PSMA aptamer conjugated to DNP (PSMA-DNP) or control aptamer conjugated to DNP (Ctrl-DNP), incubated with $Alexa_{647}$ labeled anti-mouse IgG and analyzed by flow cytometry (n=1). **b**. C57BL/6 mice vaccinated against DNP (DNP-KLH) or mock vaccinated (KLH) were implanted subcutaneously with 10^5 ΔPSMA expressing B16.F10 melanoma cells. Five days later 500 pmole of PSMA-DNP conjugate was administered via tail vein injection and repeated two additional times at three days interval. Tumor volume is shown at day 14 post tumor implantation (p=0.0079). **d**. Mice vaccinated as in panel C were injected intravenously with $5x10^4$ B16.F10 tumor cells and five days later 500 pmole of PSMA-DNP conjugated was administered via tail vein injection and repeated two additional times at three days interval. 28 days post tumor injection when mice in the mock vaccinated group exhibited signs of morbidity, mice were sacrificed and lung metastasis was measured by determining lung weight (p=0.0238). (5 mice/group) (n=2). Data are represented as mean ± SEM.

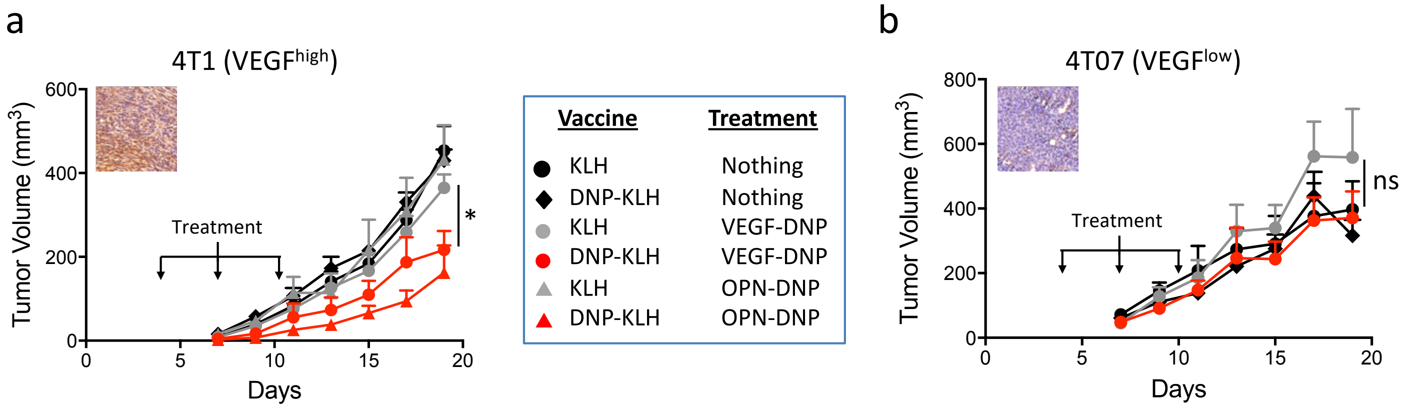

Supplementary Figure 2. VEGF-DNP and OPN-DNP conjugate mediated tumor inhibition is abrogated in 4T07 tumor cells expression low levels of VEGF or OPN. DNP and mock vaccinated mice were implanted subcutaneously with 4T1 **(a)** or 4T07 **(b)** tumors and treated with VEGF-DNP or OPN-DNP conjugates as described in Figure 2 and tumor growth was monitored. Statistical analysis: **a**. VEGF-DNP in DNP-KLH versus KLH, p=0.03; OPN-DNP in DNP-KLH versus KLH, p=0.01. **b.** No significant differences among the groups. (6 mice/group) (n=2). Data are represented as mean ± SEM.

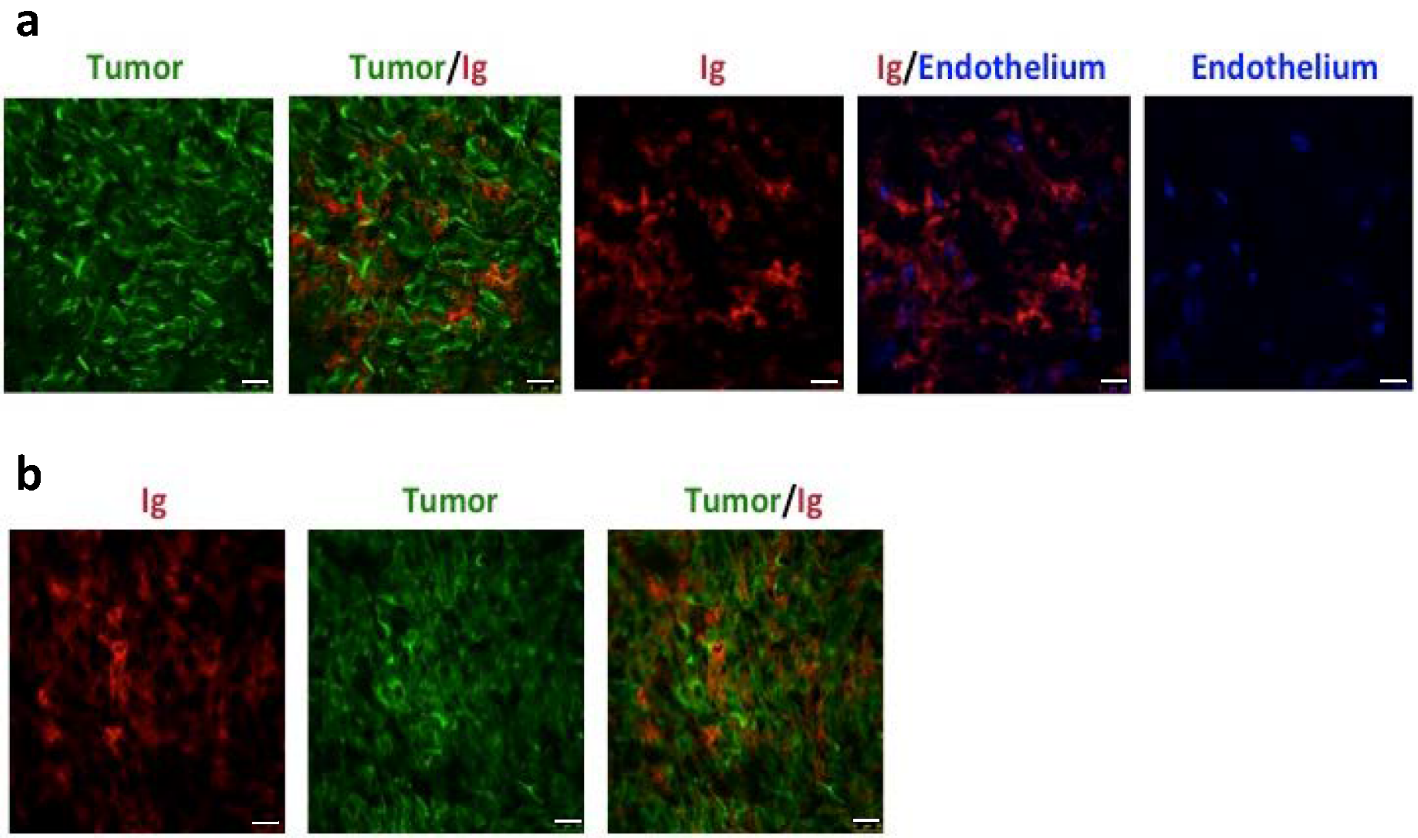

Supplementary Figure 3. Immunoglobulin deposits localize in proximity of tumor cells in DNP immunized mice treated with VEGF-DNP. Palpable 4T1 tumor bearing mice prevaccinated against DNP were injected intravenously with VEGF aptamer-DNP conjugate and 24 hours later tumors were excised. Immunohistochemistry was performed using antibodies against IgM/IgG (**red**), pan-endothelial marker (**blue**) and wide spectrum cytokeratin staining mainly the 4T1 tumor cells (**green**). Tissue sections were imaged using a Leica SP5 inverted confocal microscope with a 40x HCX PL APO/1.25 objective as described. Shown in **a** and **b** are tumors from two different mice. Individual channels and merge of Ig and Tumor (**a** and **b**) and Ig and Endothelium (**a**). Scale bar = 25 μm. *(n=2)*

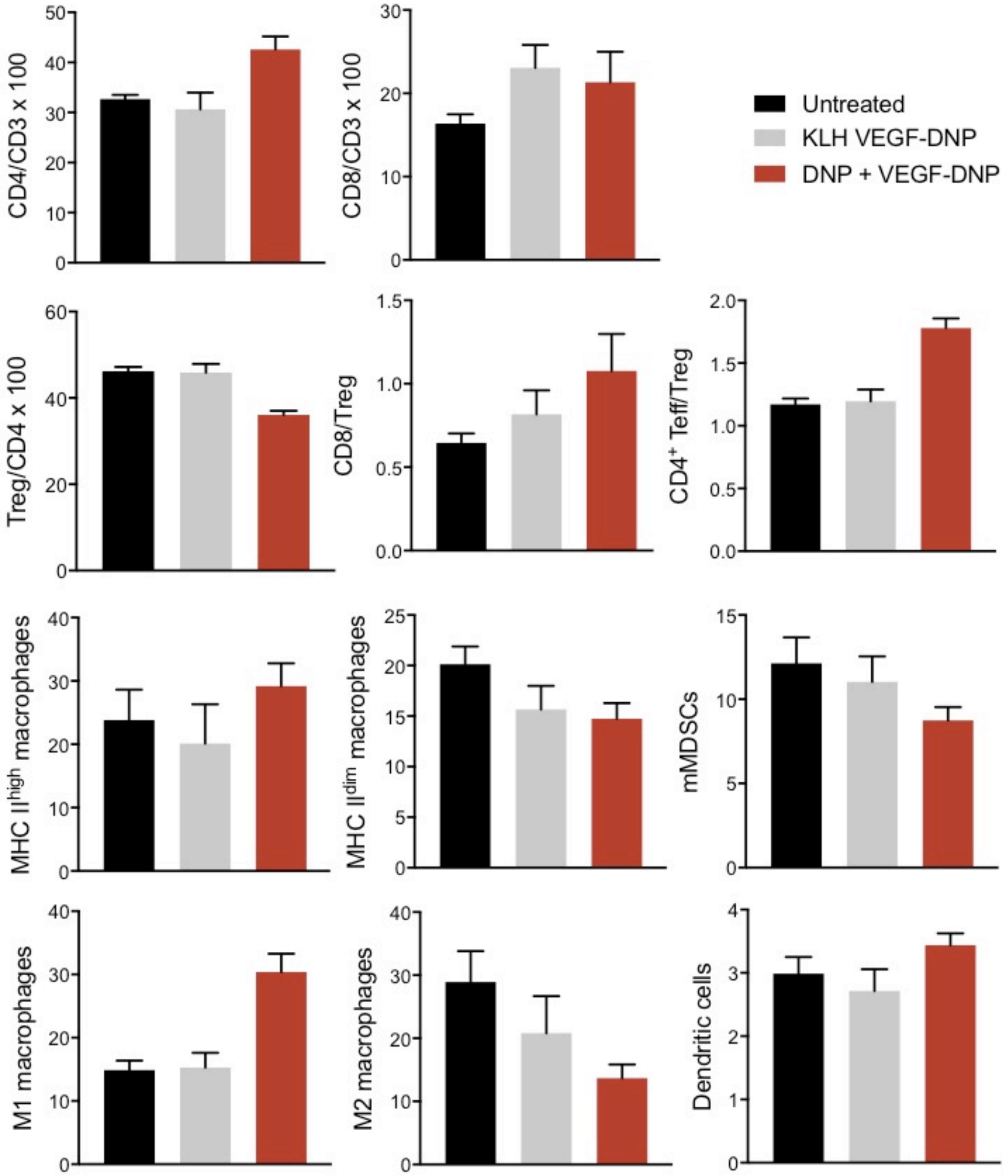

Supplementary Figure 4. Infiltration of immune subsets in DNP (DNP) and control (KLH) vaccinated mice challenged with 4T1 tumors and treated with VEGF-DNP conjugates as described in Figure 2. Tumors were harvested 2 days following the last treatment and cell suspensions were prepared for multiparameter flow cytometry analysis (Methods). Macrophage subsets, MDSC, and DC, are presented as percent of $CD45^+$ cells. Treg, $CD4^+CD25^+Foxp3^+$; MHC $II^{high \& low}$ macrophages: $Ly6C^-F4/80^+$; M1 macrophages: $F4/80^+CD11c^{low}$; M2 macrophages: $F4/80^+CD11c^{high}$; MDSC, $Ly6C^+F4/80^-$; DC: $CD11c^+$MHC II^+.(n=1). Data are represented as mean ± SEM.
